# Prognosis and Risk Factors of Stroke After Thoracic Endovascular Aortic Repair for Stanford Type B Aortic Dissection

**DOI:** 10.3389/fcvm.2021.787038

**Published:** 2022-01-10

**Authors:** Zhengbiao Zha, Youmin Pan, Zhi Zheng, Xiang Wei

**Affiliations:** Division of Cardiothoracic and Vascular Surgery, Tongji Hospital, Tongji Medical College, Huazhong University of Science and Technology, Wuhan, China

**Keywords:** stroke, risk factors, thoracic-endovascular procedures, Stanford B type aortic dissection, aortic dissection

## Abstract

**Background:** Stroke is a severe complication of patients with type B aortic dissection (TBAD) after thoracic endovascular aortic repair (TEVAR). Our aim is to identify predictors of stroke after TEVAR.

**Methods:** From February 2016 to February 2019, 445 patients with TBAD who underwent TEVAR were retrospectively analyzed. Univariate and multivariate analyses were performed to identify predictors of stroke after TEVAR.

**Results:** The total incidence of stroke was 11.5%, with transient neurological dysfunction (TND) of 10.6% and permanent neurological dysfunction (PND) of 0.9%. The average age of the patients was 53.0 ± 3.2 years, and the male/female ratio was 1.17. Univariate analysis suggested that age, body mass index (BMI), diabetes mellitus, chronic obstructive pulmonary disease (COPD), the urgency of repair, type of anesthesia, and left subclavian artery (LSCA) processing were potential risks factors of stroke after TEVAR. Multiple logistic regression identified that LSCA coverage (OR = 5.920, 95% CI: 2.077–16.878), diabetes mellitus (OR = 3.036, 95% CI: 1.025–8.995), and general anesthesia (OR = 2.498, 95% CI: 1.002–6.229) were independent predictors of stroke after TEVAR.

**Conclusions:** Left subclavian artery (LSCA) coverage, diabetes mellitus, and general anesthesia were independent risk factors of stroke after TEVAR for TBAD.

## Introduction

Since Dake's first description of thoracic endovascular aortic repair (TEVAR) in 1994, TEVAR has gained widespread acceptance and serves as the preferred treatment for patients with type B aortic dissection (TBAD). TEVAR has been associated with decreased short-term morbidity and mortality, but stroke remains its major debilitating complication (1). Stroke often leads to higher early mortality, longer hospital stays, and a poor quality of life. Stroke is maybe a consequence of thromboembolism as aortic wall atheroembolism dislodged by wire or catheter manipulation (2, 3) or low-flow brain ischaemia following coverage of the left subclavian artery (LSCA) with insufficient collateral circulation. Debates on the etiology of stroke after TEVAR are still going on. Therefore, our aim is to identify the modifiable risk factors of stroke in these patients.

## Materials and Methods

### Patients

The study was a retrospective review and approved by our internal review board. From February 2016 to February 2019, 467 patients with TBAD who underwent TEVAR were retrospectively obtained from medical records of Tongji Hospital of Huazhong University of Science and Technology. Aortic dissection (AD) was classified according to the Stanford classification. The main TEVAR indications were as follows: refractory pain or refractory hypertension; acute cerebral, visceral, or lower extremity malperfusion; high-risk radiographic features (maximal aortic diameter over 4 cm, proximal primary entry tears over 1 cm, prone to aortic rupture or progression, maximal aortic diameter over 6.5 cm or diameter increments more than 1 cm per year for chronic aortic dissection, etc.). Cases with isolated thoracic aortic lesions were included in this study. Patients who were treated for thoracic-abdominal aneurysms, concomitant abdominal aneurysms, or requiring the deployment of a stent-graft across the left common carotid artery (LCCA) were excluded (18 with abdominal aneurysms, four with stent-graft across LCCA). Finally, 445 cases were eligible in our research. Among these eligible cases, 203 had acute type B aortic dissection (<2 weeks after symptom onset), two suffered from the prior transient ischaemic attack, nine had a history of lacunar infarction, 11 suffered from mild carotid artery atherosclerosis and stenosis, three had symptoms of intestinal ischaemia, 23 suffered from acute lower extremity malperfusion, and no patients with symptoms of cerebral malperfusion. When patients were planning an LSCA coverage, preoperative computed tomography angiography (CTA) of the head and neck was performed to evaluate the posterior cerebral circulation. Patient demographics, previous medical history, preoperative status, and intraoperative and postoperative data were reviewed and recorded. Patients were followed up either by telephone or by visiting the outpatient clinic for a duration of 30 days after TEVAR. Patients who had a stroke within 30 days, even if discharged, were arranged a consultation with our experienced neurologist and received a cost-free brain imaging check if the diagnosis is necessary. CTA examinations of the aorta were routinely performed during the 30-day follow-up.

### Procedure

#### TEVAR

In brief, the stent-graft procedures were performed in a digital subtraction angiography (DSA) suite. Heparin sodium of 70 U/kg was administered intravenously. A 5-French angiographic pigtail catheter was advanced into the ascending aorta to permit an arteriographic evaluation of the distance between the LSCA, the entry tear, and the diameter of the aorta where the stent-graft will be implanted. A 22–24 French sheath was introduced transfemorally over a 0.035-in stiff guidewire. The stent-graft was then delivered through the sheath and placed within the true lumen of the aorta. After the stent-graft was deployed, arteriography was performed to confirm the position of the device relative to the entry tear and to evaluate the size of and flow within the aortic lumens and branch vessels. Two types of the thoracic stent graft, Hercules thoracic stent graft (Microport Medical, Shanghai, China) and Relay thoracic stent graft (Bolton Medical, Sunrise, FL), were used in these patients.

#### LSCA Processing

Prophylactic LSCA revascularization was performed, when patients were prepared for LSCA coverage with an incomplete circle of Willis, a dominant left vertebral artery, or the upper limb ischaemia. LSCA coverage without revascularization was applied to achieve a more extensive proximal seal when patients have dominant right vertebral artery, developed circle of Willis, or absence of upper extremity ischaemia.

Surgical bypass from LCCA to LSCA, served as our first bypass choice, was performed by central approach. Briefly, a transverse supraclavicular skin incision of 6 to 8 cm was made across the two heads of the sternocleidomastoid muscle, the platysma muscle was divided, then the internal jugular vein was exposed between the two muscle heads. Medial to this vein, LCCA was exposed by opening the carotid sheath. When the scalene fat pad was mobilized laterally from the LCCA, LSCA was then exposed beneath the anterior scalene muscle. Next, the origin of LSCA was ligated to prevent type II endoleaks. Finally, an 8 to 10 mm Dacron prosthesis was anastomosed side to side to bypass from LCAA to LSCA through a subcutaneous tunnel.

When LCAA was not suitable for bypass (atherosclerosis, stenosis, or dissection), an alternative bypass from the right axillary artery (RAA) to the left axillary artery (LAA) was performed by bilateral infraclavicular approach. Briefly, a transverse lateral infraclavicular skin incision of about 4 cm was made, and the incision was deepened through the subcutaneous tissue and the pectoral fascia, then the area lateral to the major pectoral muscle and medial to the minor pectoral muscle was exposed. In this area, there was a fatty pad where the AA lay in the depths. Finally, an 8 to 10 mm Dacron prosthesis was anastomosed side to side to bypass from RAA to LAA through a subcutaneous tunnel. The Amplatzer vascular plugs (AGA Medical Corp, Plymouth, Minn) were used to prevent type II endoleaks and maintain the antegrade vertebral artery flow if necessary.

#### Definiitions

The primary endpoint of this study was any postoperative stroke within 30 days. Stroke was defined as the presence of neurological symptoms and signs such as motor, sensory, or cognitive dysfunction persisting for ≥24 h and clinical suspicion of stroke. The stroke was divided into permanent neurological dysfunction (PND) and transient neurological dysfunction (TND). PND was defined as postoperative coma, ischaemic hypoxic encephalopathy, cerebral infarction or hemorrhage, or other structural injuries confirmed by means of computed tomographic scanning or MRI of the brain. TND was defined as postoperative confusion, seizure, agitation, or delirium with no detected structural abnormality in the brain and symptoms relief occurred usually before hospital discharge. All neurologic complications were reviewed with a neurologist experienced in the perioperative care of this patient population. Emergency surgery was defined as an operation performed within 24 h after admission. Acute renal dysfunction was defined as an estimated glomerular filtration rate <60 ml/min/1.72 m^2^ or dialysis. Refractory hypertension was defined as hypertension persisting despite more than three different classes of antihypertensive medications at maximal recommended or maximally tolerated doses. Malperfusion was defined as inadequate blood flow to a tissue bed. The transient ischaemic attack was defined as a transient episode of neurologic dysfunction caused by focal brain, spinal cord, or retinal ischaemia without acute infarction. Other definitions were referred to the Society for Vascular Surgery (SVS) and Society of Thoracic Surgeons (STS) reporting standards for TBAD (4).

#### Statistics Analysis

Statistical analysis was performed using the SPSS 22.0 statistical software (IBM Corporation, Armonk, NY, USA). Continuous data were expressed as mean ± SD. Categorical variables were shown as frequencies with corresponding percentages. Differences between groups were tested by means of the chi-square test or Fischer's exact test, as appropriate. The student's *t*-test or Mann-Whitney U test was used to compare continuous variables. In univariate analysis, categorical data were analyzed by chi-square or Fisher exact tests, and continuous data were analyzed by using *t*-test or Wilcoxon rank sum tests. Forward stepwise multivariate logistic regression analysis was performed to determine the independent predictors of stroke. All variables suggested by the univariate analysis (*p* < 0.2) or those judged to be clinically important were entered into the logistic regression models. Statistical significance was defined as *p* < 0.05 and all results were two-tailed.

## Results

### Patient Characteristics

Among these patients, stroke occurred in 11.5% (51/445) patients, including PND (0.9%; 4/445) and TND (10.6%; 47/445). Among the patients with PND, three suffered from severe cerebral hemorrhages and one had a massive cerebral infarction. Post-operation brain imaging showed that PND lesion in one patient was in the posterior circulation territory and both the anterior and posterior circulation territories in the other three patients. One hemorrhage case might be secondary to anticoagulant treatment for acute cerebral infarction, and the other two cases were maybe due to hypertensive cerebral hemorrhage. Patients with TND displayed symptoms such as confusion, seizure, agitation, delirium, or mixed transient neurological symptoms and signs, with no detected structural abnormality in the brain after imaging scanning. The average age was 53.0 ± 3.2 years and the male/female ratio was 1.17. Patients with diabetes were associated with an increased stroke rate compared with diabetes-free patients (22.6 vs. 10.6%, *p* = 0.044). Fifteen cases were found with atherosclerotic plaques in the aortic arch at preoperative CTA imaging. These patients with plaques seemed to have a higher stroke rate than patients without aortic arch plaques, but had no significant statistical difference between the two groups (20.0 vs. 11.2%, *p* = 0.520). Eleven of 15 aortic arch plaque cases had concomitant diabetes; there was a strong link between diabetes and aortic arch plaque (*r* = 0.536, *p* = 0.00000000). Patients who suffered from stroke were more often above the age of 65 years and with concomitant obesity, diabetes, and chronic obstructive pulmonary disease (COPD). Details are listed in Table 1.

**Table 1:** Demographic characteristics and preoperative data of patients.

**Variables**	**Total**	**Stroke**	**Non-Stroke**	***P***
	**(*n* = 445)**	**(*n* = 51)**	**(*n* = 394)**	
Age (year)	53.0 ± 3.2	53.6 ± 3.5	53.0 ± 3.1	0.186
<55	334 (75.1%)	38 (74.5%)	296 (75.1%)	
55–65	104 (23.4%)	11 (21.6%)	93 (23.6%)	
≥65	7 (1.6%)	2 (3.9%)	5 (1.3%)	
Male	240 (53.9%)	29 (56.9%)	211 (53.6%)	0.665
BMI (kg/m^2^)	23.5 ± 5.3	24.7 ± 5.4	23.4 ± 5.3	0.092
<24	256 (57.5%)	28 (54.9%)	228 (57.9%)	
24–30	131 (29.4%)	15 (29.4%)	116 (29.4%)	
≥30	58 (13.0%)	8 (15.7%)	50 (12.7%)	
Smoking	131 (29.4%)	16 (31.4%)	115 (29.2%)	0.747
Hypertension	240 (53.9%)	29 (56.9%)	211 (53.6%)	0.655
Diabetes mellitus	31 (7.0%)	7 (13.7%)	24 (6.1%)	0.044
Hyperlipidaemia	230 (51.7%)	27 (52.9%)	203 (51.5%)	0.849
COPD	20 (4.5%)	5 (9.8%)	15 (3.8%)	0.052
PVD	104 (23.4%)	11 (21.6%)	93 (23.6%)	0.747
CVD	12 (2.7%)	2 (3.9%)	10 (2.5%)	0.566
Aortic arch plaque	15 (3.4%)	3 (5.9%)	12 (3.0%)	0.520

*BMI, Body mass index; COPD, chronic obstructive pulmonary disease; PVD, peripheral vascular disease; CVD, cerebral vascular disease*.

A total of 337/445 (75.7%) of the patients accepted general anesthesia by patients' self-selection and had a higher stroke rate compared with patients under local anesthesia (13.4 vs. 5.6%, *p* = 0.027). A total of 226/445 (50.8%) patients underwent coverage of LSCA without revascularization, 124/445 (27.8%) accepted coverage of LSCA with revascularization, and the remaining patients have LSCA remained uncovered. Stroke rates were 1.6% for patients with LSCA coverage and revascularization, 5.3% for patients whose LSCA remained uncovered, and 19.5% for patients with LSCA coverage without revascularization. LSCA coverage without revascularization was associated with a higher stroke rate, in comparison with LSCA revascularization combined with remaining uncovered (19.5 vs. 3.2%, *p* = 0.00000007). Among the 124 revascularizations of LSCA patients, 85 patients accepted LSCA fenestration, 39 patients underwent LSCA bypass revascularization (5 RRA to LRA bypass with one using Amplatzer vascular plugs for the LSCA origin occlusion, and 34 LCCA to LSCA with all ligation of the origin), and there was no significant difference between two procedures in stroke rate (1.2 vs. 2.6%, *p* = 0.583).

A total of 153/445 (34.4%) patients accepted emergency or urgent operations. The average operation time was 196 ± 45.2 min and the average hospital stay was 8 ± 3.8 days. The total 30-day mortality rate was 1.1% (5/445); two died of sudden aortic rupture and three died of multiple organ dysfunction syndrome (MODS). Patients with stroke had higher mortality than the others (3.9 vs. 0.8%, *p* = *0*.044). Type I/III endoleaks were observed in four cases during the 30-day follow-up period. These patients were left untreated but followed up closely. Eleven patients experienced acute renal failure and recovered after active therapy at the intensive care unit. Twenty-five patients experienced pulmonary infection and three of them died of MODS resulting from severe drug-resistant bacteria infection. Patients with stroke were more susceptible to pulmonary infection (31.4 vs. 2.3%, *p* = 0.0000000). Details are listed in Table 2.

**Table 2:** Operating data and early outcomes.

**Variables**	**Total**	**Stroke**	**Non-Stroke**	***P***
	**(*n* = 445)**	**(*n* = 51)**	**(*n* = 394)**	
Emergency or urgent	153 (34.3%)	23 (45.1%)	130 (33.0%)	0.087
General anesthesia	337 (75.7%)	45 (88.2%)	292 (74.1%)	0.027
LSCA processing				0.000
Un-coverage	95 (21.3%)	5 (9.8%)	90 (22.8%)	
Coverage	226 (50.8%)	44 (86.3%)	182 (46.2%)	
Revascularization	124 (27.9%)	2 (3.9%)	122 (31.0%)	
Operation time (min)	196 ± 45.2	201 ± 47.8	196 ± 44.8	0.415
Hospital stay (days)	8 ± 3.8	11 ± 5.4	8 ± 3.4	
ICU stay (days)	4 ± 2.0	5 ± 1.9	4 ± 2.0	
**Early events**				
30-day mortality	5 (1.1%)	2 (3.9%)	3 (0.8%)	
Type I/III endoleak	4 (0.9%)	0 (0.0%)	4 (1.0%)	
Stroke	51 (11.5%)	~	~	
TND	47 (10.6%)	~	~	
PND	4 (0.9%)	~	~	
Acute Kidney Injury	11 (2.5%)	2 (3.9%)	9 (2.3%)	
Pulmonary infection	25 (5.6%)	16 (31.4%)	9 (2.3%)	

*TND, Transient neurological dysfunction; PND, permanent neurological dysfunction; LSCA, left subclavian artery*.

### Logistic Regression

Among the preoperative and intraoperative variables, age, body mass index (BMI), diabetes mellitus, COPD, general anesthesia, emergency urgency of repair, and LSCA processing (LSCA remained uncovered, coverage without revascularization, coverage but revascularization) were the difference between stroke (+) and non-stroke (–) groups (*p* < 0.2) in univariate analysis. After multivariate logistic regression analysis, LSCA coverage (*p* = 0.001), diabetes mellitus (*p* = 0.045), and general anesthesia (*p* = 0.050) were identified as independent predictors of stroke after TEVAR for TBAD, and LSCA coverage was the strongest predictor with an OR of 5.920. Details are listed in Table 3.

**Table 3:** Multivariate logistic regression for stroke after thoracic endovascular aortic repair for Stanford type B aortic dissection.

**Variables**	**B**	***P*-Value**	**Odds Ratio 95% CI**
General anesthesia	0.915	0.050	2.498 (1.002–6.229)
LSCA processing			
Coverage	1.778	0.001	5.920 (2.077–16.878)
Revascularization	−0.860	0.325	0.423 (0.076–2.342)
Diabetes mellitus	1.1111	0.045	3.036 (1.025–8.995)
Age	0.027	0.570	1.028 (0.936–1.128)
BMI	0.033	0.268	1.034 (0.975–1.096)
COPD	0.992	0.132	2.696 (0.742–9.798)
Emergency or urgent	0.510	0.113	1.666 (0.886–3.135)

*LSCA, left subclavian artery; BMI, body mass index; COPD, chronic obstructive pulmonary disease*.

## Discussion

Stroke is a devastating complication of TBAD after TEVAR, which is maybe a consequence of thromboembolism or hypo-perfusion after coverage of the aortic arch branch vessels with insufficient collateral circulation. In our study, most cases (47/51) had a mild stroke after TEVAR, and ischaemic stroke might be accounted for approximately 94.1% (48/51) of these patients. Since it was hard to distinguish the stroke types (ischaemia or hemorrhage, TND or PND), we combined all strokes together to enhance statistical sensitivity. The main finding of our study was that LSCA coverage, diabetes mellitus, and general anesthesia were independent risk factors of stroke after TEVAR for TBAD.

Left subclavian artery (LSCA) coverage is often necessary to achieve proximal seal in up to 40% of patients treated with TEVAR (5). In our study, 50.8% of patients who accepted LSCA coverage without revascularization got an increased stroke rate, compared with coverage with revascularization or LSCA uncovered. Similarly, other researchers found the risk of neurologic complications increased after coverage of the LSCA after TEVAR (6, 7). LSCA coverage leads to reduced or obstructed blood flow to the left vertebral artery, which theoretically can provoke cerebrovascular ischaemia and stroke (8). Additionally, coverage of the more proximal aortic arch requires delivery of bulky stent-grafts across the orifice of arch vessels, potentially leading to embolization into the cerebral circulation. Conversely, some researchers considered that there were no significant associations between LSCA coverage and stroke (9–11). We noticed that mild cerebral infarction was not included in the above research, which could be the reason why we got different conclusions. Other recent meta-analyses demonstrated that LSCA revascularization did lower the stroke incidence after TEVAR, with pooled stroke rates of 3.2% for LSCA remaining uncovered, 5.3% for coverage with revascularization, and 8% for coverage without revascularization (12). We are of the same opinion. The stroke probably increases if the LSCA is covered during the TEVAR, particularly in those without revascularization. Moreover, we found that the two ways to reconstruct the blood flow of the LSCA, LSCA fenestration and bypass, are similar in lowering the stroke incidence after TEVAR. LSCA revascularization should be beneficial for patients with TEVAR at risk.

The etiology of a stroke may have other different causes. Diabetes has the pathophysiological characteristics of inflammation, proliferation, and hypercoagulability. Diabetes is associated with higher stroke risk compared with diabetes-free persons (13, 14). Diabetes causes various microvascular and macrovascular disorders often culminating in major clinical complications. Individuals with diabetes are especially susceptible to the consequences of cerebral small vessel diseases. Moreover, atherosclerotic plaques may be deposited in the aortic arch and arch branch vessels. Although the presence of these plaques does not necessarily induce stroke, strokes may occur when plaque is dislodged by excessive intravascular manipulations during TEVAR, which is intrinsic to the procedure. We considered the stroke incidence to be greatly increased after TEVAR in patients with diabetes, particularly in those who had poor cerebral vascular reserve capacity and unstable aortic arch plaques. Therefore, controlling diabetes (diet, insulin, plaque stabilization with statins, etc.) was important to reduce the incidence of stroke in patients who underwent TEVAR.

Our study also showed that the probability of stroke in patients who underwent TEVAR after general anesthesia would significantly increase, mostly presented as TND, such as delirium, which is a neurobehavioural syndrome caused by the transient disruption of normal neuronal activity secondary to systemic disturbances. Because the etiology of stroke after TEVAR was hardly distinguished, we did not rule out the patient's delirium only due to general anesthesia, but it is also an important stroke event that we are concerned about and should be avoided as much as possible. General anesthesia may interfere with the body's physiological homeostasis and increase the risk of stroke. As an alternative method, whenever the patients' condition permits, it should be encouraged to apply local anesthesia for patients with TEVAR at risk. Under local anesthesia, new percutaneous TEVAR can be performed safely and effectively by two Proglide vascular closure systems (Abbott Vascular, SantaClara, Calif), while maintaining a lower risk of access-related complications (15). Therefore, our data advocated for applying local anesthesia for high-risk stroke TEVAR patients.

The present study still had several limitations. First, the data were collected retrospectively, and potential selection bias in our population could not be completely avoided. Second, our patients simply came from a single center, although the sample size was large enough; the follow-up period was not long; patients may not have a stroke during the perioperative period but suffer from a delayed stroke during the long-term follow-up, which we have to pay attention to; and our future research will amend the follow-up data. Finally, anesthesia level during operation may affect our result, but we consider the main reason should be the patient's own reasons and the TEVAR operation itself, which may be an open question to answer, although we applied neurobiological monitor during the procedure.

In conclusion, the stroke rate (11.5%) after TEVAR in our investigation is somehow relatively high, but most cases were mild and transient strokes with no detected brain structural abnormality. We found LSCA coverage, diabetes mellitus, and general anesthesia were independent risk factors of stroke after TEVAR for TBAD. So, applying the LSCA revascularization, local anesthesia, as well as diabetes treatment would reduce the incidence of stroke after TEVAR.

## Data Availability Statement

The raw data supporting the conclusions of this article will be made available by the authors, without undue reservation.

## Ethics Statement

The studies involving human participants were reviewed and approved by Tongji Hospital of Huazhong University of Science and Technology. Written informed consent for participation was not required for this study in accordance with the national legislation and the institutional requirements. Written informed consent was not obtained from the individual(s) for the publication of any potentially identifiable images or data included in this article.

## Author Contributions

ZZha performed the conception and design of the manuscript, the patient data acquisition and analysis, and the draft of the article. YP, ZZhe, and XW helped with the design of the manuscript, the draft, and revised the article. All authors have read and approved the final manuscript.

## Conflict of Interest

The authors declare that the research was conducted in the absence of any commercial or financial relationships that could be construed as a potential conflict of interest.

## Publisher's Note

All claims expressed in this article are solely those of the authors and do not necessarily represent those of their affiliated organizations, or those of the publisher, the editors and the reviewers. Any product that may be evaluated in this article, or claim that may be made by its manufacturer, is not guaranteed or endorsed by the publisher.
